# Effective distance of volatile cues for plant–plant communication in beech

**DOI:** 10.1002/ece3.7990

**Published:** 2021-08-02

**Authors:** Tomika Hagiwara, Masae Iwamoto Ishihara, Junji Takabayashi, Tsutom Hiura, Kaori Shiojiri

**Affiliations:** ^1^ Department of Agriculture Ryukoku University Otsu Japan; ^2^ Graduate School of Agriculture Kyoto University Kyoto Japan; ^3^ Ashiu Forest Research Station Field Science Education and Research Center Kyoto University Kyoto Japan; ^4^ Center for Ecological Research Kyoto University Otsu Japan; ^5^ Department of Ecosystem Studies Graduate School of Agricultural and Life Sciences The University of Tokyo Tokyo Japan

**Keywords:** beech, effective distance, *Fagus crenata*, induced defense, plant–plant communication, volatile organic compounds

## Abstract

In response to volatiles emitted from a plant infested by herbivorous arthropods, neighboring undamaged conspecific plants become better defended against herbivores; this is referred to as plant‒plant communication. Although plant‒plant communication occurs in a wide range of plant species, most studies have focused on herbaceous plants. Here, we investigated plant‒plant communication in beech trees in two experimental plantations in 2018 and one plantation in 2019. Approximately 20% of the leaves of a beech tree were clipped in half in the spring seasons of 2018 and 2019 (clipped tree). The damage levels to leaves in the surrounding undamaged beech trees were evaluated 90 days after the clipping (assay trees). In both years, the damage levels decreased with a reduction in the distance from the clipped tree. In 2019, we also recorded the damage levels of trees that were not exposed to volatiles (nonexposed trees) as control trees and found that those that were located <5 m away from clipped trees had significantly less leaf damage than nonexposed trees. By using a gas chromatograph–mass spectrometer, ten and eight volatile compounds were detected in the headspaces of clipped and unclipped leaves, respectively. Among them, the amount of (*Z*)‐3‐hexenyl acetate in clipped leaves was significantly higher than that in nonclipped leaves. Our result suggests that green leaf volatiles such as (*Z*)‐3‐hexenol and (*Z*)‐3‐hexenyl acetate and other volatile organic compounds emitted from clipped trees induced defenses in the neighboring trees within the 5 m radius. The effective distances of plant‒plant communication in trees were discussed from the viewpoint of the arthropod community structure in forest ecosystems.

## Graphical abstract

To disclose the effective distance for plant–plant communication through volatiles is important to understand not only arthropod community but also ecosystem, because plant's qualities change after receiving volatiles. Therefore, we investigated communication between beech trees through volatile in the field and determined the effective distance.

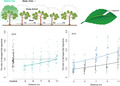

## 1 INTRODUCTION

Plants emit volatile organic compounds (VOCs) in response to leaf damage caused by herbivorous arthropods or mechanical damage such as weeding (Takabayashi & Shiojiri, 2019). When neighboring plants receive such VOCs, they often induce defenses against herbivores; this phenomenon is referred to as plant–plant communication (Heil & Bueno, 2007; Heil & Karban, 2010; Karban et al., 2000, 2014; Shiojiri & Karban, 2008b; Tscharntke et al., 2001; Yoneya & Takabayashi, 2014). For example, field‐grown sagebrush trees (*Artemisia tridentata*) which were exposed to VOCs from clipped conspecific plants in the early season experienced less herbivore damage in the following seasons (Shiojiri & Karban, 2008a). Lima bean (*Phaseolus lunatus*) plants exposed to VOCs from herbivore‐damaged conspecific plants increased the amount of extrafloral nectar that attracts carnivorous arthropods (Choh et al., ,^2004^, ^2006^; Choh & Takabayashi, 2006; Heil & Kost, 2006). Plant–plant communication mediated by VOCs has been observed in >40 plant species, most of which are herbaceous (Heil & Karban, 2010; Karban et al., 2014; Li, 2016; Yoneya & Takabayashi, 2014). However, the first evidence of the phenomenon was found in tree species such as sugar maple and poplar (Baldwin & Schultz, 1983).

Besides laboratory or greenhouse conditions (e.g., Arimura et al., 2004; Girón‐Calva et al., 2014), plant–plant communication in trees under field conditions has been reported in only six taxa: poplar (*Populus euramericana*) (Frost, Mescher, Dervinis, et al., 2008), willows (*Salix sitchensis* and *S. eriocalpa*) (Pearse et al., 2013; Yoneya & Takabayashi, 2013), black alder (*Alnus glutinosa*) (Dolch & Tscharntke, 2000; Tscharntke et al., 2001), birch (*Betula* spp.) (Himanen et al., 2010), and sagebrush (*Artemisia tridentata*) (Karban et al., 2006); these taxa are either early‐successional tree species or shrub species. Late‐successional species dominate forests for a long time and provide important ecological and socioeconomic value. However, to the best of our knowledge, there has been no plant–plant communication studies in late‐successional tree species.

Determining the effective distance of VOCs may be important for understanding arthropod communities because the quality of leaves directly affects arthropod distribution. The effective distance has only been studied in two species (black alder and sagebrush) under field conditions. Karban et al. (2006) revealed that the effective distance of sagebrush (*Artemisia tridentata*) was 0.6 m by comparing leaf damage among control and exposed plants. In black alder, the number of damaged leaves increased with increasing distance (up to 10 m) from a manually defoliated conspecific tree (Dolch & Tscharntke, 2000). The number of specialist herbivores of black alder (i.e., leaf beetles [*Agelastica alni*]) was higher on the farthest tree (10.6 m from the defoliated tree) than on the nearest tree (1.3 m from the defoliated tree) (Tscharntke et al., 2001). Furthermore, leaf beetles avoided leaves from the nearest alder tree and preferred those from the farthest tree for both feeding and oviposition in laboratory experimental assays. Although these authors focused on the distance of plant–plant communication in alder trees, they did not compare the damage of exposed trees to that of nonexposed trees. Therefore, the effective distance for plant‒plant communication in forests remains unknown.

Japanese beech (*Fagus crenata* Blume) (hereafter “beech”) is a late‐successional tree species that often dominates cool‐temperate mesic forests in Japan (Hiura, 1995). The dominant herbivorous arthropods are beech caterpillars (*Quadricalcarifera punctatella*) and gypsy moth (*Lymantria dispar*) larvae (Nakamura et al., 2014). Damage caused by these herbivores increases the C:N ratio and the tannin and phenolic compound contents in beech leaves (Aoyama & Koike, 2011; Kamata, 1996). Manual leaf‐clipping reduces the leaf nitrogen content (Kamata et al., 1996). These studies showed that beech can induce direct defenses against herbivory. Beech also exhibits an induced defense via VOCs, whereby undamaged beech leaves respond to VOCs from clipped leaves within the same tree (intraplant signaling), causing systemically induced resistance (Hagiwara & Shiojiri, 2020). However, to the best of our knowledge, volatiles of damaged beech have not yet been identified. Since plant–plant communication via volatiles from damaged plants is hypothesized to originate from intraplant signaling (Heil & Bueno, 2007), we hypothesize that undamaged beech trees can eavesdrop on damage‐induced volatiles from damaged trees (interplant signaling).

The objective of the present study was to clarify whether beech trees exhibit plant–plant communication under field conditions and to determine the effective distance. We also analyzed the VOCs between damaged and undamaged beech. Finally, we discuss the effect of plant–plant communication in beech on the arthropod community in a forest ecosystem.

## 2 MATERIAL AND METHODS

### 2.1 Beech plantations

Field experiments were conducted in beech plantations in Tomakomai Experimental Forest (42°7′N, 141°6′E, 220 m a.s.l.), Hokkaido University (hereafter called Tomakomai), in 2018 and 2019, and in Hiruzen Experimental Forest (35°3′N, 133°6′E, 510 m a.s.l.), Tottori University (hereafter called Hiruzen), in Japan in 2018. These two plantations were both established in 1991 by planting beech seedlings that were approximately 4 years old, the seeds of which were obtained from a variety of sources (Osada et al., 2018). The beech seedlings were planted on flat land at Tomakomai and on a slope facing west in Hiruzen. In both plantations, beech trees reached heights of 10–13 m. Leaf expansion began in early May and leaf abscission began in mid‐October.

### 2.2 Experimental design

**FIGURE 1:**
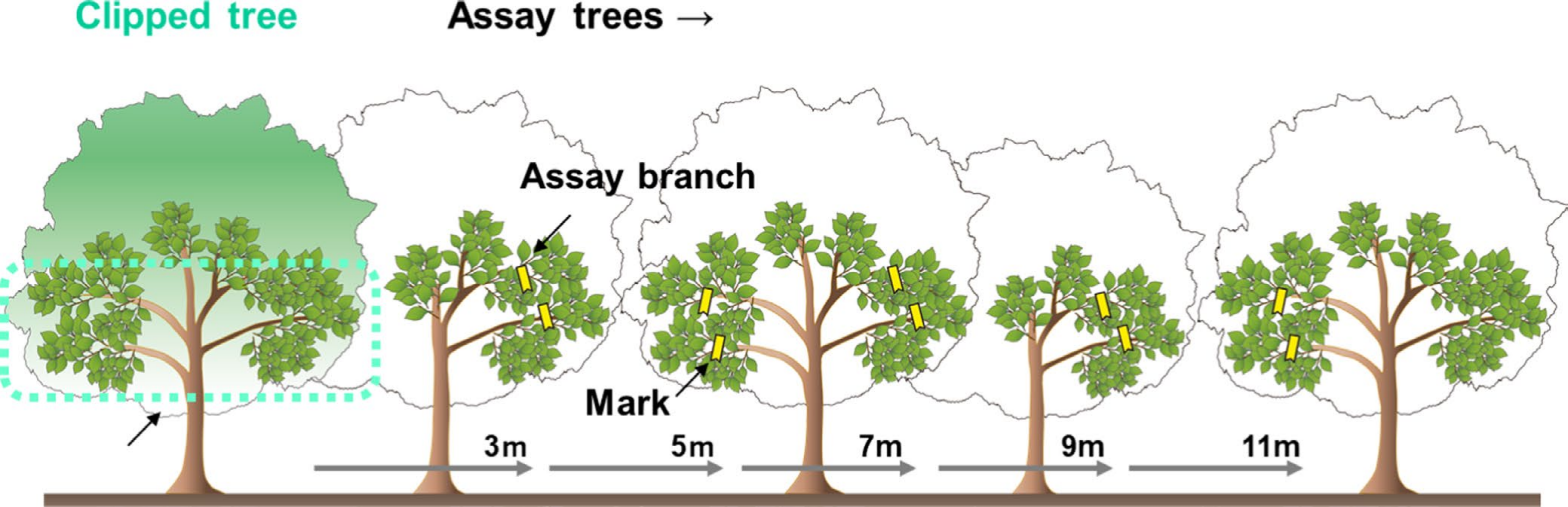
Schematic diagram of experimental designs. Twenty percent of leaves were cut into half by scissors in each clipped tree. The assay branches were selected and marked from other trees (assay trees)

**FIGURE 2:**
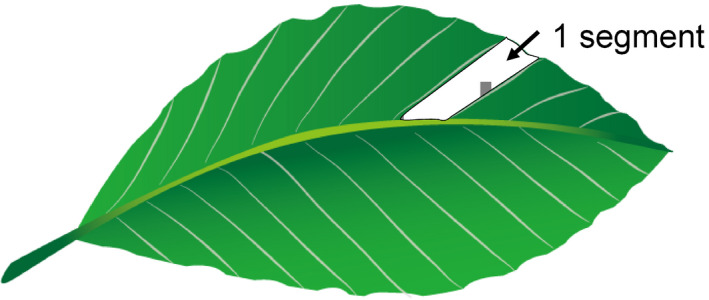
Each leaf segment was a portion of the leaf blade that was surrounded by parallel leaf veins

We selected three trees in Tomakomai and two trees in Hiruzen as volatile‐emitting trees in 2018. We clipped approximately 20% of the leaves of each tree into halves (hereafter called clipped trees). We conducted the clipping on May 22 in Tomakomai and June 12 in Hiruzen when herbivore species were active. The surrounding undamaged beech trees were assigned as volatile‐exposed trees (assay trees). In each assay, 10 branches with 8–12 leaves were marked on trees that were located 3, 5, 7, 9, or 11 m from a clipped tree. We repeated the experiments three times in Tomakomai and twice in Hiruzen (Figure 1). When a tree covered two distance levels (e.g., 5 m and 7 m; Figure 1), we selected 10 branches for each distance. After 90 days, the number of leaves damaged by chewing herbivores and pathogens was recorded for each branch. As beech leaves consist of segments (Figure 2), we evaluated the level of damage caused by herbivores by counting the total number of segments damaged by herbivores and pathogens in a leaf. Each leaf segment was a portion of the leaf blade that was surrounded by parallel leaf veins (Figure 2). Beech leaves were damaged by *Actias aliena*, *Sphrageidus similis*, and other herbivores as well as yellow leaf spot (Wei & Harada, 1998) and other leaf spots caused by pathogens.

To evaluate the effective distance of the plant–plant communication, we compared the damage levels of unexposed beech (control trees) with those of 13 assay trees in 2019 at Tomakomai. The experimental conditions in 2019 were the same as those in 2018. Clipping was performed on May 31. From the 2018 experiment, we found that leaf damage increased as the distance from the clipped tree increased until 7–11 m. Among 7‐, 9‐, and 11‐m branches, the deference either is or not significantly different. This result means that the control treatment needs to be >11 m away. We assayed 15 trees which were planted 13.0–66.5 m from clipped trees as controls and selected 93 sample branches from these trees on May 31. After 90 days, the number of leaf segments damaged by herbivores and pathogens was recorded for each branch, as in 2018.

### 2.3 Chemical analysis of headspace VOCs from beech leaves

We collected VOCs under field conditions in Tomakomai. The sampling field was different from the field in which the exposure experiments were conducted. Beech seeds were collected in the area of the Hakodate Forestry Office in 1973. To reveal the differences in VOCs between the clipped and control treatments, we randomly selected eight beech trees. On 10 and 11 June 2019 we tightly enclosed a branch with approximately 30 undamaged leaves in a PTS bag (500 mm × 350 mm; Mitsubishi Gas Chemical Co. Tokyo, Japan). Using Tenax TA as an adsorbent, the VOCs in the bag were collected for 60 min at an airflow rate of 100 ml/min. Airflow was generated using an air pump (Sibata Scientific Technology Ltd., Saitama, Japan). After collecting VOCs from the undamaged leaves, we clipped approximately 50% of the leaves of the branch in half using scissors. After clipping, we re‐enclosed the branch to collect VOCs from clipped leaves using the same procedure.

The VOCs trapped on Tenax TA were eluted with 2 ml of diethyl ether (FujifilmWako Pure Chemical, Osaka, Japan). We injected a 1‐μl aliquot of the eluate into the injection port (250℃) of a gas chromatograph–mass spectrometer (GC‒MS: QP2010SE, Shimadzu, Kyoto, Japan) equipped with HP‐5MS capillary column (0.25 mm i.d., length 30 m, film thickness 0.25 µm; Agilent Technologies, Santa Clara, CA, USA). The oven temperature of the GC‒MS was programmed to increase from 40℃ (5 min hold) to 280℃ at a rate of 10℃ min^‐1^. Detected compounds were tentatively identified by comparison with mass spectra in the Wiley 7 N database. VOCs were further identified by comparing their mass spectra with those of the authentic compounds. The total ion intensities were used to compare the amount of each compound between the undamaged and clipped conditions.

### 2.4 Statistical analyses

To test whether the leaf damage of assay trees changed along the distance from clipped trees in the 2018 experiment, we used a generalized linear mixed effect model (GLMM) which applied the Laplace approximation and maximum likelihood via the lmer function in the lme4 package of R 4.0 (R Core Team, 2020). All statistical tests were performed using R software. Since the response variable (i.e., the total number of damaged leaf segments of a branch) was a discrete variable, we fitted GLMMs with a Poisson distribution and a logit‐link function. Since some branches were sampled from the same tree, we used the mixed effect model to account for nonindependent errors due to nested measurements (Faraway, 2016). Since the number of segments per leaf differed among trees, the mean of the total number of segments of a branch was included in the model as an offset term. The full model included the following: distance, plantation, and the interaction term between distance and plantation as fixed effects; and tree as a random effect. To test the effect of distance and plantation, we conducted chi‐squared tests between the full and reduced models.

To estimate the effective distance of plant–plant communication, we applied the GLMM for the 2019 experiment. The full model included distance as a fixed effect and tree as a random effect; this model was tested by chi‐squared tests against the null model without distance. To further estimate the effective distance, data of branches at a 3‐m distance from neighboring trees were combined with those of control trees. The GLMM model with the treatment (control and assay) as a fixed effect and tree as a random effect was applied. This model was tested by the chi‐squared tested against the model without treatment, assuming no difference between the control and 3‐m distance neighboring assay trees. This process was sequentially repeated for 5‐, 7‐, 9‐, and 11‐m distances. Some trees were used for clipping experiments in both 2018 and 2019. Our results and conclusion did not change even if 2‐year data were analyzed together incorporating the repeated measurements by GLMM (result not shown). To compare VOCs between the control and clipped treatments, we conducted a paired *t*‐test for each compound.

## 3 RESULTS

### 3.1 Effective distance of plant–plant communication

**FIGURE 3:**
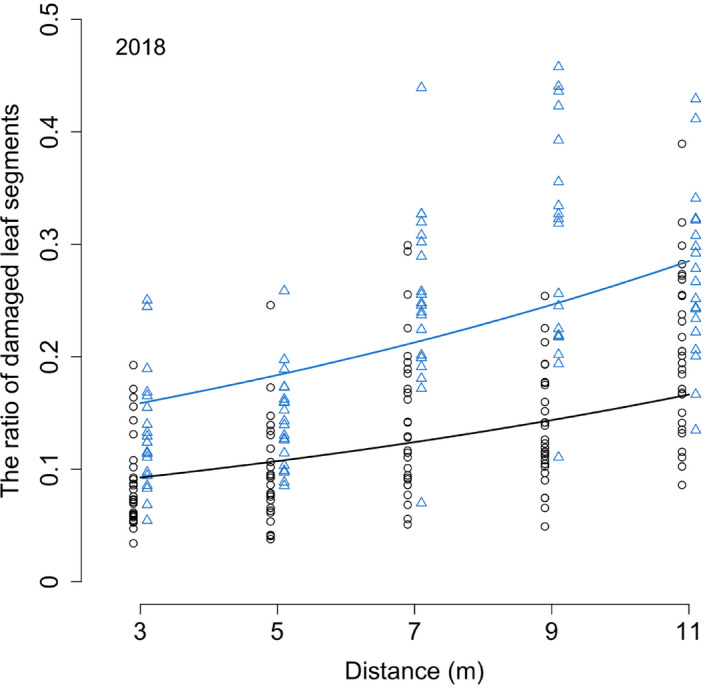
The changes in ratio of the damaged leaf segments along the distance from clipped trees. The circles are the ratio of the damaged leaf segments to total leaf segments of each sampled branch in Tomakomai (black circle) and in Hiruzen (blue triangle). Two curves show the estimated values by GLMM for Tomakomai (black line) and Hiruzen (blue line), respectively

For the 2018 data, the full model with distance, plantation, and the interaction between distance and plantation (Distance × Plantation) as fixed effects was compared with reduced models. Reduced models were the models assuming the effect of distance and plantation (Distance +Plantation), effect of distance alone (Distance), and difference between plantations (Plantation); and the null model assumed no difference between plantation and distance (Null). The most‐fit models included distance and plantation as fixed effects in 2018 (Table 1). Damaged leaf segments increased with distance from clipped trees. In other words, trees neighboring clipped trees showed less damage than those located at long distances from the clipped trees (Figure 3). The leaf damage level in Hiruzen was almost twice as high as that in Tomakomai at each distance in 2018.

**TABLE 1:** Summary of generalized linear mixed effect model (GLMM) for the number of damaged segments

Fixed effects of candidate models	Parameter	AIC	LL	Deviance	*df*	Chisq	*p*
2018							
Distance + Plantation + Distance × Plantation	5	2,616.5	−1,303.3	2,606.5			
**Distance + Plantation**	**4**	**2,616.7**	**−1,304.4**	**2,608.7**	**1**	**2.2**	.**1386**
Distance	3	2,629.0	−1,311.5	2,623.0	1	14.3	.0002
Plantation	3	2,680.8	−1,337.4	2,674.8	1	66.1	<.0001*
Null	2	2,687.2	−1,341.6	2,683.2	1	8.4	.0037
2019							
**Distance**	**3**	**1,416.0**	**−704.98**	**1,410.0**			
Null	2	1,436.4	−716.19	1,432.4	1	22.4	<.0001

Note

Shown are number of parameters used in the model (Parameter), Akaike's Information Criterion (AIC), log‐likelihood (LL), difference in degree of freedom against above model (*df*), Chi‐squared value (Chisq), *p*‐value (*p*).

The random effect was not shown.

*Chi‐square test against the model with Distance + Plantation.

**FIGURE 4:**
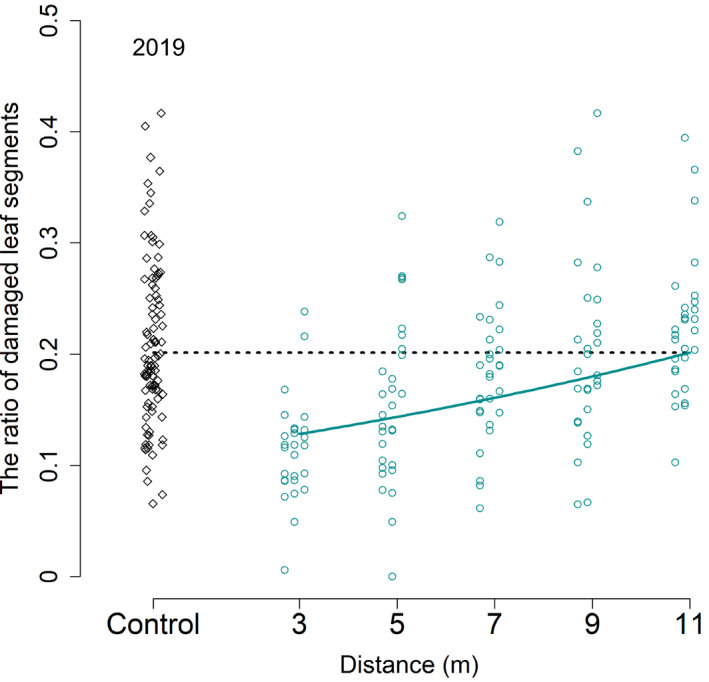
Comparison of the assay and control treatment in the ratio of the damaged leaf segments. The circles are the ratio of the damaged leaf segments to total leaf segments of each assay branch. The diamonds show the ratio of the damaged leaf segments to total leaf segments of each control branches. Continuous and dotted lines show the estimated ratio by GLMM for the assay and control branches, respectively

For the 2019 data, the full model with distance as a fixed effect was compared to the null model assuming no effect of distance (Null). Similar to 2018, the effect of distance was significant, and the nearer neighbors of the clipped tree showed less leaf damage than the farther trees (Table 1; Figure 4).

Assay trees located up to a 5‐m distance from clipped trees showed significantly lower leaf damage than control trees (Table 2; *p* = .002963). Assay trees at a 7‐m distance showed a trend (Table 2; *p* = .1080). The difference between the damage level of assay and control trees gradually decreased as the distance from the clipped trees increased. Assay trees at distances of 9 and 11 m from clipped trees showed leaf damage levels similar to those in control trees.

**TABLE 2:** Summary of chi‐squared test in comparison with control and the exposed trees

Distance from the volatile source trees	*df*	Chisq	*p*
2019			
3 m	1	20.1670	.000007
5 m	1	8.8300	.002963
7 m	1	2.5827	.108000
9 m	1	1.0095	.315000
11 m	1	0.2889	.590900

Note

Summary of Chi‐squared test for the difference between the control and assay trees at each distance in Tomakomai in 2019. For each distance, the model with the treatment (assay and control) as a fixed effect was compared to the model without treatment assuming no difference between assay and control trees (null model).

### 3.2 Chemical analysis of headspace VOCs of beech leaves

We detected 10 principal compounds in the control and clipped leaves (Table 3). The amounts of released (*Z*)‐3‐hexenol and (*Z*)‐3‐hexenyl acetate were significantly different between the control and clipped leaves (*t* = −3.2345, *df* = 7, and *p* = .01436; and *t* = −2.8892, *df* = 7, and *p* = .02334, respectively). These two compounds represented >50% of the total VOCs obtained from the clipped leaves. Caryophyllene alcohol was not detected in the control leaves; however, it was not significantly different between the control and clipped leaves (*t* = −0.88352, *df* = 7, and *p* = .4063). The variance was large for (*Z*)‐3‐hexenol, (*Z*)‐3‐hexenyl acetate, (+)‐.δ.‐Cadinene, α.‐Calacorene, Caryophyllenyl alcohol, and α.‐Cadinol. Sabinene, the principal monoterpene found in European beech (*Fagus sylvatica* L.) (Dindorf et al., 2006), was not detected in Japanese beech.

**TABLE 3:** Amounts of volatile compounds from control beech and clipped beech

Compound name	Amount (peak areas (×10^4^)/g)	
	Control	Clipped
(*Z*)‐3‐hexenol	n.d	911.3 ± 281.7*
(*Z*)‐3‐hexenyl acetate	532.3 ± 277.4	3,962.3 ± 1,069.5*
Limonene	85.2 ± 30.2	80.7 ± 19.5
γ. Muurolene	112.1 ± 21.3	176.2 ± 46.6
α.‐Muurolene	120.3 ± 14.9	121.9 ± 38.7
(+)‐.δ.‐Cadinene	221.2 ± 44.5	317.7 ± 104.8
α.‐Calacorene	26.3 ± 17.2	16.8 ± 11.5
Caryophyllenyl alcohol	n.d	73.8 ± 37.3
α.‐Cubeben	54.5 ± 20.6	83.4 ± 30.1
α.‐Cadinol	181.5 ± 44.5	364.8 ± 110.4

Note

Data are the peak areas (×10^4^) of total ion chromatograms per dry leaf weight (g) (mean ± *SE*). n.d. indicates that these compounds were not detected. Asterisks indicate significant differences by paired *t*‐test at *p* < .05.

## 4 DISCUSSION

In both plantations, leaf damage decreased in unclipped trees in close proximity to the clipped trees compared to far‐neighboring unclipped trees (Figure 3; Table 1) or control trees (Figure 3; Tables 1 and 2). The damage level differed between plantations (Figure 3), which may be because Hiruzen was in the natural range of beech, while Tomakomai was outside its natural range (Matsui et al., 2004), and because the herbivore communities may have differed between the two sites (Nakamura et al., 2014). We also found considerable variation in the damage level among samples within the same plantation, which may be due to variations in the seed origin. Notably, even though our experiment may have included such variations in herbivore communities and beech individuals as in natural ecosystems, we found a significant decrease in leaf damage in nearby neighboring trees.

After herbivore damage or manual clipping, plants release VOCs, such as monoterpenes, sesquiterpenes, and green leaf volatiles (GLVs) (Li, 2016). We found considerable variation in the volatiles emitted by individual beech. Among these compounds, (Z)‐3‐hexenol and (*Z*)‐3‐hexenyl acetate (which are categorized as GLVs) were the two most common volatile compounds emitted by the clipped leaves (Table 3). Shiojiri et al. (2006) demonstrated that one of the ecological functions of GLVs is defense against herbivores and pathogens in *Arabidopsis thaliana*. GLVs are also known to activate defense in various plant species and are a highly conserved type of signaling molecules (Li, 2016; Yamauchi et al., 2015). After exposure to (*Z*)‐3‐hexenyl acetate (i.e., a GLV), poplar began priming their defenses prior to experiencing any physical damage (Frost et al., 2008). GLVs are very rapidly produced and emitted upon herbivory or pathogen infection by almost every green plant, playing a crucial role in plant defense (Scala et al., 2013). These studies showed that GLVs induced the defense of intact plants. Our results show that GLVs and other VOCs emitted from clipped beech induced the defense of neighboring beech against herbivores and/or pathogens, resulting in reduced damage to neighboring beech. Intraplant signaling for plant defense against herbivore has previously been reported in late‐successional tree species (Hagiwara & Shiojiri, 2020). However, to the best of our knowledge, our study is the first to show conspecific plant–plant communication in late‐successional tree species in the field.

Assay trees located up to 5 m from clipped trees showed significantly less leaf damage than control trees. As the distance from the clipped trees increased, the leaf damage between the assay and control trees gradually became similar. Therefore, we suggest that the effective distance of plant communication in beech is 5–7 m under our experimental conditions. However, the effective distance may be farther if the effect of induced VOC interactions between undamaged plants (Arimura et al., 2004). Previous studies have shown effective distances of 0.6 m in sagebrush (*Artemisia tridentata*) (Karban et al., 2006) and ≤10 m in black alder (Dolch & Tscharntke, 2000). Evidently, the exact value of the effective distance may vary depending on the tree size, damage intensity, and tree species. Furthermore, effective distance may be affected by herbivore abundance and spatial distribution. If herbivore density is low and the distribution is highly localized, plants that eavesdropped the VOCs may not respond easily because the response itself entails certain costs. Plants may have evolved so that only near neighboring trees respond, and the effective distance may be short. On the contrary, if herbivores outbreak at long distances, the effective distance may be far. Karban et al. (2013) suggested that whether or not plants respond after eavesdropping depends on the kin recognition of trees; if so, the effective distance may differ depending on whether the emitter is related. VOC composition may also differ between conspecific trees and may relate to the genetic relatedness of trees (Ishizaki et al., 2011; Karban et al., 2013; Hiura et al., 2021; Shiojiri et al., 2021). Further studies are needed regarding the effective distance of VOCs.

Plant defenses induced via plant–plant communication may affect arthropod community composition, abundance, and spatial distribution in forest ecosystems. Tscharntke et al. (2001) showed that the number of phytophagous nonspecialists and specialists did not differ prior to the experiment in early May. However, 81 days after the treatment, the nonspecialists did not show a significant pattern, whereas the specialists exhibited a clear difference depending on the distance from the volatile source alder tree. In the present study, we evaluated the damage caused by either folivorous insects, leaf mining insects, sucking insects, or pathogens on a segment which were assigned to be damaged. Future studies to record the damage for each feeding guild are required to clarify the effective distance for each feeding guild.

We found that the effective distance of plant signaling in beech was 5–7 m under our experimental conditions. These beech heights were 10–13 m, that is, half the size of trees in the natural beech forest (Tateishi et al., 2010). Indeed, the effective distance was farther in the natural beech forest than in our study. Although plant–plant communication via volatiles has been mostly studied in herbaceous species, it is also necessary to study such signaling in tree species. Since trees (especially late‐successional species) dominate large areas for long time periods with large biomass, plant–plant communication in trees may have a significant effect on herbivory and pathogen abundance in large forest landscapes. VOC emissions from trees contribute largely to atmospheric VOCs (Matsunaga et al., 2009; Mentel et al., 2013; Šimpraga et al., 2019); therefore, VOC emissions due to herbivory damage may affect atmospheric VOCs. Consideration of the effective distance of damage‐related VOCs may be important for further understanding the spatial distribution of herbivore communities and forest ecosystem functions.

## CONFLICT OF INTEREST

The authors declare that there are no conflicts of interest.

## AUTHOR CONTRIBUTIONS

**Tomika Hagiwara:** Conceptualization (lead); data curation (equal); formal analysis (lead); funding acquisition (equal); investigation (lead); methodology (equal); project administration (equal); writing‐original draft (equal); writing‐review & editing (supporting). **Masae Iwamoto Ishihara:** Formal analysis (lead); software (equal); supervision (equal); writing‐review & editing (equal). **Tsutom Hiura:** Data curation (supporting); formal analysis (supporting); project administration (supporting); writing‐review & editing (supporting). **Junji Takabayashi:** Conceptualization (supporting); supervision (supporting); writing‐review & editing (equal). **Kaori Shiojiri:** Conceptualization (lead); data curation (equal); formal analysis (supporting); investigation (equal); methodology (equal); project administration (lead); supervision (equal); writing‐original draft (equal); writing‐review & editing (equal).

## Data Availability

Data from this manuscript were archived in the publicly accessible repository Dryad (https://doi.org/10.5061/dryad.9w0vt4bf2).
